# Photon-counting distributed free-space spectroscopy

**DOI:** 10.1038/s41377-021-00650-2

**Published:** 2021-10-12

**Authors:** Saifen Yu, Zhen Zhang, Haiyun Xia, Xiankang Dou, Tengfei Wu, Yihua Hu, Manyi Li, Mingjia Shangguan, Tianwen Wei, Lijie Zhao, Lu Wang, Pu Jiang, Chengjun Zhang, Lixing You, Leigang Tao, Jiawei Qiu

**Affiliations:** 1 School of Earth and Space Science, University of Science and Technology of China, 230026 Hefei, China; 2 School of Atmospheric Physics, Nanjing University of Information Science & Technology, 210044 Nanjing, China; 3 Hefei National Laboratory for Physical Sciences at the Microscale, 230026 Heifei, China; 4 Changcheng Institute of Metrology & Measurement, Aviation Industry Corporation of China, 100095 Beijing, China; 5 State Key Laboratory of Pulsed Power Laser Technology, National University of Defense Technology, 230037 Hefei, China; 6 State Key Laboratory of Marine Environmental Science, College of Ocean and Earth Sciences, Xiamen University, 361102 Xiamen, China; 7 Shanghai Institute of Microsystem and Information Technology, Chinese Academy of Sciences, 200050 Shanghai, China

**Keywords:** Imaging and sensing, Optical sensors, Near-infrared spectroscopy

## Abstract

Spectroscopy is a well-established nonintrusive tool that has played an important role in identifying and quantifying substances, from quantum descriptions to chemical and biomedical diagnostics. Challenges exist in accurate spectrum analysis in free space, which hinders us from understanding the composition of multiple gases and the chemical processes in the atmosphere. A photon-counting distributed free-space spectroscopy is proposed and demonstrated using lidar technique, incorporating a comb-referenced frequency-scanning laser and a superconducting nanowire single-photon detector. It is suitable for remote spectrum analysis with a range resolution over a wide band. As an example, a continuous field experiment is carried out over 72 h to obtain the spectra of carbon dioxide (CO_2_) and semi-heavy water (HDO, isotopic water vapor) in 6 km, with a range resolution of 60 m and a time resolution of 10 min. Compared to the methods that obtain only column-integrated spectra over kilometer-scale, the range resolution is improved by 2–3 orders of magnitude in this work. The CO_2_ and HDO concentrations are retrieved from the spectra acquired with uncertainties as low as ±1.2% and ±14.3%, respectively. This method holds much promise for increasing knowledge of atmospheric environment and chemistry researches, especially in terms of the evolution of complex molecular spectra in open areas.

## Abstract (web summary)

A photon-counting distributed free-space spectroscopy is proposed and demonstrated using lidar technique. It is suitable for remote spectrum analysis with range resolution over wide band.

## Graphical abstract

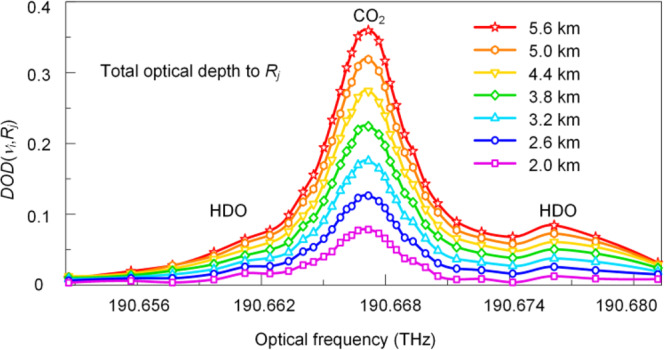

## Introduction

Molecular spectroscopy dates back to Newton’s division of sunlight into a color bar from red to violet using a prism. With the advent of quantum mechanics and lasers, the precise spectral analysis method has developed rapidly in many fundamental domains, with applications ranging from the quantum description of the matter to non-intrusive diagnostics of various media. On the one hand, accurate and sensitive spectroscopic absorption measurements are achieved in the laboratory via different methods, such as cavity ring-down spectroscopy^1^, intracavity laser absorption spectroscopy^2^, and optical comb spectroscopy^3,4^ (Michelson-comb spectroscopy^5^, dual-comb spectroscopy^6–8^, and Ramsey-comb spectroscopy^9^). On the other hand, greenhouse gases and atmospheric pollutants are monitored in the atmosphere by various techniques, such as grating spectrometers^10^, Fourier transforms spectroscopy^11–13^, and differential optical absorption spectroscopy^14,15^. As an example, dual-comb spectroscopy is applied for spectra analysis in a high-finesse optical cavity^7^, and in an open path between a telescope and retroreflectors installed at different ranges^16,17^. The approaches mentioned above provide either in-situ or column-integrated spectral information. However, molecular characteristics vary rapidly in time and space due to chemical reactions and physical transportation in free atmosphere. It would be beneficial to analyze the processes continuously with range-resolved spectra in open areas, especially in inaccessible regions.

Lidar techniques sense three-dimensional distributions of molecules remotely^18,19^. Raman scattering lidar and differential absorption lidar (DIAL) are two representative lidar techniques. The former is based on the inelastic Raman scattering with several orders of magnitude lower efficiency than that of elastic scattering, which is appropriate for measuring molecules with high concentrations^20^. The latter emits two lasers at different frequencies alternatively. The intensity of laser at online frequency is strongly absorbed by the molecule under investigation, and that at nearby offline frequency is weakly absorbed^21,22^. The two-frequency DIAL is optimized for specific molecule detection thus not suitable for multi-gas analysis, lacking the full information of different gas absorption lines. Instead of acquiring the differential absorption in two frequencies, multi-frequency DIAL obtains the entire absorption spectrum by emitting laser pulses at dozens of frequencies, where frequency accuracy is a challenge. Frequency-locking techniques are demonstrated, including gas cell-referenced scanning lasers^23,24^, phase-locked laser diodes^25^, and frequency-agile optical parametric oscillators^26^. Usually, the integral spectra along the optical path are obtained in these multi-frequency DIALs, at the sacrifice of range resolution.

Here, photon-counting distributed free-space spectroscopy (PDFS) is proposed based on lidar techniques. Range-resolved optical spectrum analysis is achieved along the outgoing laser beam. In order to realize wideband frequency scanning and locking in lidar for the analysis of multiple gases spectra, the comb-referenced locking method^27–29^ is preferred rather than the traditional gas cell-referenced locking method^23,24^. Firstly, the frequency of an external cavity diode laser (ECDL) is stabilized by locking it to an optical frequency comb via heterodyne detection. Secondly, to suppress the fluctuation in coupling efficiency of telescope caused by turbulence, a superconducting nanowire single-photon detector (SNSPD) with a large-active area is manufactured. The SNSPD, with high detection efficiency, low dark noise, and broadband response is a promising detector for infrared remote sensing^30–34^. Thanks to the high signal-to-noise ratio (SNR) offered by the SNSPD, one can implement range-resolved spectrum analysis over a large optical span (~100 nm), using a low-power fiber laser. Thirdly, during the time-consuming detection process (usually a few minutes, since photon accumulation is used to enhance the SNR), aerosol loading variation, detector efficiency drift, and laser power fluctuation may introduce unexpected errors. To deal with these problems, a reference laser at a fixed frequency or a scanning probe laser fires alternately (employing time-division multiplexing technique), implementing differential absorption detection at each scanning step. Therefore, the laser pulses share the same acoustic-optical modulator (AOM), amplifier, telescope, optical path in space, SNSPD, and electric circuits, making the optical layout simple and robust thus free of repeated calibration. In other words, the proposed system can be regarded as an integrated system of dozens of two-frequency DIALs. It naturally holds much potential for greenhouse gas monitoring, leakage warning, and atmospheric chemistry researches.

The concentration of carbon dioxide (CO_2_) in the atmosphere has increased rapidly since the industrial age. Accurate assessment of carbon dioxide emissions is important to project future climate change^35^. At present, the carbon emissions peak and carbon neutrality are among the most concerning topics of discussion worldwide. Here, multi-dimensional analyses of atmospheric CO_2_ and HDO are demonstrated, which means one can analyze the gas in the time-range-spectrum domain in free atmosphere. By combining the analyses with real-time meteorological conditions, the continuous diurnal variation of CO_2_ and HDO over time, as well as the carbon capture capability of plants over range can be clearly observed.

## Results

### Principle and experimental set-up

In the selection of a suitable absorption line for CO_2_, the temperature insensitivity and the strength of the gas absorption line are key factors that influence the precision and sensitivity of the measurement^36,37^. The CO_2_ R16 line at 190.667 THz shows temperature insensitivity with a ground state energy of 106.130 cm^−1^ and a relatively higher line strength of 1.779 × 10^−23^ cm^−1^/(molecule cm^−2^) than other CO_2_ lines in the C and L bands^38^. An optical spectrum range of 190.652–190.682 THz is chosen, which covers the CO_2_ R16 line and also contains two weak semi-heavy water (HDO, isotopic water vapor) lines (Fig. 6b). The detailed analysis of absorption line selection is provided in Supplementary information.

**Fig. 1:**
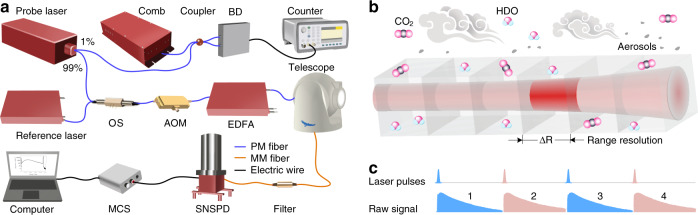
Optical layout. **a** Experimental set-up. The detailed parameters of the instruments are listed in Table S2. BD balanced detector, OS optical switch, AOM acousto-optic modulator, EDFA erbium-doped fiber amplifier, SNSPD superconducting nanowire single-photon detector, MCS multi-channel scaler, PM polarization-maintaining, MM multi-mode. **b** Light propagating in the atmosphere. The path length in the red sections represents the range resolution of ∆*R*, and the spectra within this range in the whole free space can be obtained. **c** Time sequence of the time-division multiplexing technique

A diagrammatic view of PDFS is shown in Fig. 1. A tunable ECDL (Toptica, CTL1550) covering 185.185–196.078 THz is used as a probe laser. One percent of the probe laser is split out and combined with an optical frequency comb for heterodyne detection, offering an accurate frequency reference when tuning the frequency of the probe laser (for details, see Fig. 6 in the section “Materials and methods” section). The other 99% of the probe laser and a reference laser (Rayshining, PEFL) are chosen alternatively by using a fast optical switch (OS) and chopped into laser pulses via an AOM, with a pulse repetition rate of 20 kHz and a full width at half maximum (FWHM) of 400 ns. The pulse energies of chopped pulses are amplified via an erbium-doped fiber amplifier (EDFA) to 40 μJ. Such an optical layout ensures that the probe pulse and the reference pulse have exactly the same shape. The time sequence of time-division multiplexing is shown in Fig. 1c, where the probe pulse is tagged in odd numbers (1, 3, 5, etc.) and the reference pulse is tagged in even numbers (2, 4, 6, etc.). Then, the laser pulses are pointed to the atmosphere through a collimator with a diameter of 100 mm. The probe laser scans the CO_2_ and HDO absorption spectra, while the reference laser remains at a stable frequency of 190.652 THz. At each scanning step, the atmospheric backscattering signals at probe frequency and reference frequency are received and coupled into an optical fiber with a core diameter of 50 μm using a telescope with a diameter of 256 mm. Two multimode fiber (MMF) bandpass filters (37 GHz flattop bandwidth) are cascaded to suppress the solar background noise in the daytime, with a total suppression ratio of 60 dB. Finally, the signal is fed to the large active-area SNSPD. The SNSPD is divided into 9 pixels, and each pixel consists of two superconducting nanowire avalanche photodetectors, which improves the maximum count rate of the detector. The total quantum efficiency of the SNSPD array is 31.5% at 190.667 THz, and the dark count rate is below 100 per second. The count rate of the detector reaches 20 MHz with quantum efficiency higher than 30.0%. The SNSPD works in its linear operating range since the count rate is below 7 MHz in the experiment.

The atmospheric backscattering contains Mie and Rayleigh components. Note that Rayleigh scattering is inversely proportional to the fourth-order of the wavelength (~1572 nm). Thus, Mie backscattering dominates in the raw lidar signal. The photon number received in the frequency of *ν* and at a range of *R*_*j*_ is expressed as^18^

1 $$N\left( {\nu ,R_j} \right) = \frac{E}{{h\nu}}\eta _{\rm {o}}\eta _{{{\mathrm{q}}}}\frac{{A_{{{\mathrm{t}}}}}}{{R_j^2}}O\left( {R_j} \right)\frac{{c\tau }}{2}\beta \left( {\nu ,R_j} \right)T_{{{\mathrm{r}}}}^2\left( {\nu ,R_j} \right)$$

where *E* is the pulse energy, *η*_o_ is the optical efficiency of the transmitted signal, *η*_q_ is the quantum efficiency, *h* is the Planck constant, *A*_t_ is the area of the telescope, *R*_*j*_ is the range, *j* represents the index of range, *O*(*R*_*j*_) is the geometrical overlap factor, *c* is the speed of light, *τ* is the pulse duration, and *β* is the Mie volume backscattering coefficient. The range resolution Δ*R* = *cτ*/2 is limited by the laser pulse duration. The transmission term *T*_r_ is given by

2 $$T_{{{\mathrm{r}}}}\left( {\nu ,R_j} \right){{{\mathrm{ = }}}}\exp \left\{ { - {\int}_0^{R_j} {\left[ {\alpha _{{{\mathrm{a}}}}\left( {\nu ,R_j} \right) + \alpha _{{{\mathrm{m}}}}\left( {\nu ,R_j} \right)} \right]{{{\mathrm{d}}}}r} } \right\}$$

where *α*_a_ is the extinction coefficient of aerosol including the effects of scattering and absorbing. *α*_m_ = *α*_g_ + *α*_s_ is the extinction coefficient of molecules, where *α*_g_ is the absorption coefficient of the gas under investigation, and *α*_s_ represents other extinction processes of molecules.

The received photon number in the probe frequency *ν*_*i*_ (*i* = 1, 2,…, 30 being the index of the frequency steps) and the reference frequency *ν*_0_ is *N*_*i*_(*ν*_*i*_, *R*_*j*_) and *N*_0_(*ν*_0_, *R*_*j*_), respectively. Since the reference frequency *ν*_0_ is close to the probe frequency *ν*_*i*_, it is assumed that in Eqs. (1) and (2), *β*, *α*_a_, and *α*_s_, have the same values. Furthermore, the probe and reference lasers share the same AOM, amplifier, optical path, SNSPD, and electric circuits. After EDFA gain and optical response correction against frequency, the only difference between Eqs. (1) and (2) is the absorption coefficient *α*_g_ of the gas under investigation. Thus, the total differential optical depth (DOD) for a single optical path is estimated as

3 $$\begin{array}{l}{\mathrm{DOD}}\left( {\nu _i,R_j} \right) = - 0.5\ln \left[ {N_i/N_0} \right]\\ {{{\mathrm{ }}}} = {\int}_0^{R_j} {\left[ {\alpha _{{{\mathrm{g}}}}\left( {\nu _i,R_j} \right) - \alpha _{{{\mathrm{g}}}}\left( {\nu _0,R_j} \right)} \right]{{{\mathrm{d}}}}r} \end{array}$$

and the DOD within a range cell (ΔDOD) from *R*_*j*_ to *R*_*j*+1_ is expressed as

4 $${\Delta}{\mathrm{DOD}}(\nu _i) = {\mathrm{DOD}}\left( {\nu _i,R_{j + 1}} \right) - {\mathrm{DOD}}\left( {\nu _i,R_j} \right)$$

which corresponds to the range-resolved absorption spectrum over the frequency scanning range. As illustrated in Fig. 1b, the absorption spectra of gases in any distributed cell along the laser beam can be obtained.

The number of received photons is stochastic in nature because of the random photon arrival and annihilation time in this quantum-limited detection^19^. The photon counts follow the Poisson distribution and the shot-noise is the mean square of the photon counts. Thus, the SNR can be described as *N*^1/2^. According to Eq. (1), a higher pulse energy *E* and a larger effective telescope area *A*_t_ are usually used to improve the SNR. Here, higher coupling efficiency contained in *η*_o_, higher quantum efficiency *η*_q_, and lower dark noise is realized by using a large active-area SNSPD. In addition, the influence of atmospheric turbulence is suppressed by using this SNSPD with an active-diameter of 50 μm^39^.

### Data acquisition and processing

**Fig. 2:**
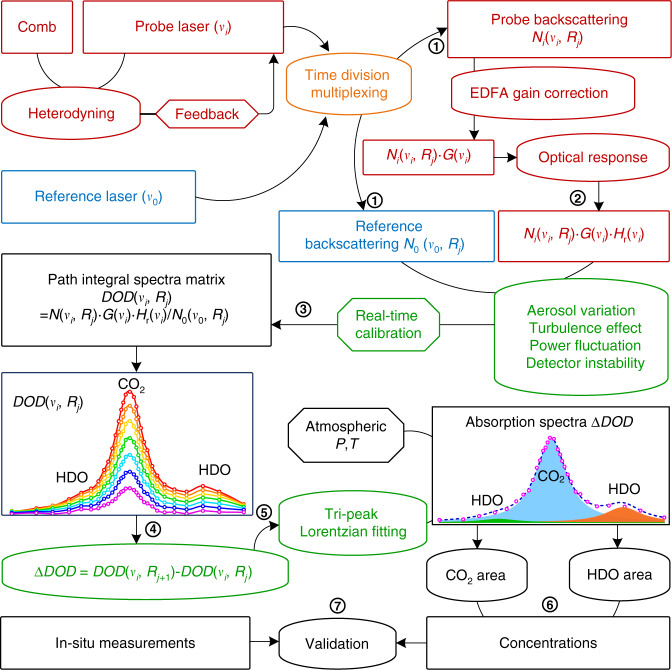
Flow chart of data acquisition and processing. ① to ⑦ represent step 1 to step 7. P pressure, T temperature

In previous work, the frequency locking technique and SNSPD with large active-area are introduced. And, the influence of HDO on CO_2_ is studied theoretically and compared experimentally in summer and winter^40^. Here, to carry out a continuous, stable, and long-range field experiment, the influence of aerosol variation, laser power fluctuation, detector instability, and signal coupling efficiency instability due to turbulence is compensated for in the data acquisition and processing process, as shown in Fig. 2.

Step 1: The backscattering profiles of the probe laser at different scanning steps and the reference laser at 190.652 THz are collected as photon counts *N*_*i*_(*ν*_*i*_, *R*_*j*_) and *N*_0_(*ν*_0_, *R*_*j*_), respectively.

Step 2: *N*_*i*_(*ν*_*i*_, *R*_*j*_) are calibrated as *N*_*i*_(*ν*_*i*_, *R*_*j*_)·*G*(*ν*_*i*_)·*H*_r_(*ν*_*i*_), considering the non-uniform optical gain of the EDFA *G*(*ν*_*i*_) and the optical response *H*_r_(*ν*_*i*_) over the scanning span.

Step 3: The aerosol variation, laser power fluctuation, detector instability, and signal coupling efficiency instability due to turbulence are calibrated by *N*_*i*_(*ν*_*i*_, *R*_*j*_)·*G*(*ν*_*i*_)·*H*_r_(*ν*_*i*_)/*N*_0_(*ν*_0_, *R*_*j*_). Thus, a matrix of DOD(*ν*_*i*_, *R*_*j*_) containing integrated absorption spectra of CO_2_ and HDO along the optical path from 0 to *R*_*j*_ is obtained.

Step 4: The range-resolved absorption spectra ΔDOD at the range *R* = (*R*_*j*_ + *R*_*j*+1_)/2 with range resolution of Δ*R* = *R*_*j*+1_−*R*_*j*_ are calculated by DOD(*ν*_*i*_, *R*_*j*+1_)−DOD(*ν*_*i*_, *R*_*j*_), where *i* changes from 1 to 30.

Step 5: Triple-peak Lorentzian nonlinear fitting is performed to separate the spectra of CO_2_ and HDO. Numerical least-squares optimization of the Lorentzian function is achieved by Levenberg–Marquardt method. Several database-based a priori constraints, including the relative strength of two HDO lines, relative frequency offsets between peaks, and the FWHM calculated with in-situ atmospheric temperature and pressure, are used in the fitting process (details are appended in the “Materials and methods” section).

Step 6: The area *A* of each separated spectrum and its standard deviation are obtained as the fitting results. Both CO_2_ and HDO concentrations and their precisions are determined by calculating the areas of the fitted spectra according to Eqs. (7)–(9).

Step 7: Concentrations are compared to the results from in-situ measurements for validation. The standard deviations between the comparisons are determined by counting the residuals of the measurements.

**Fig. 3:**
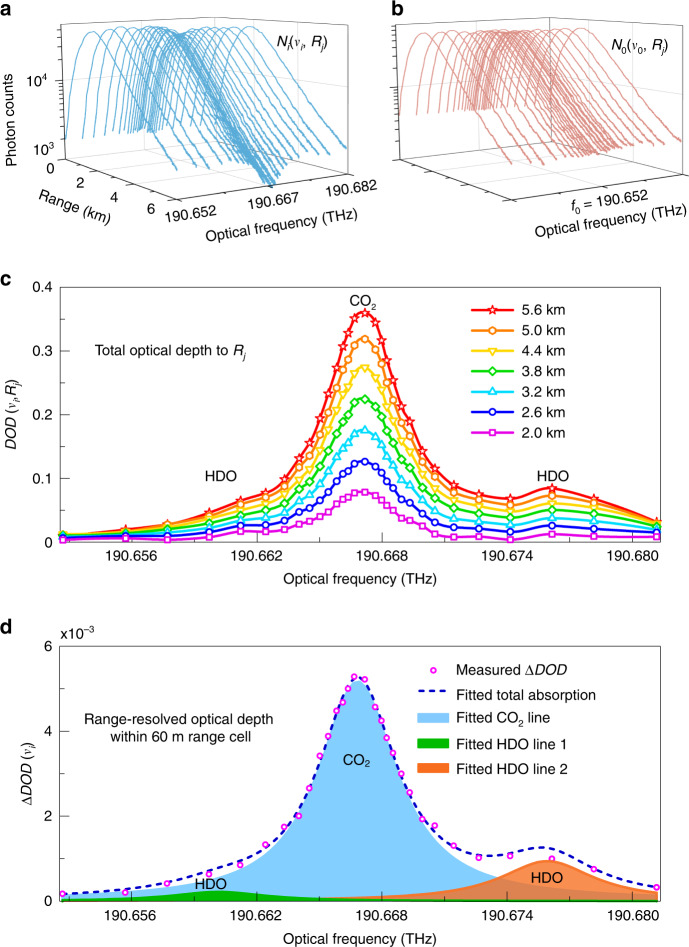
Backscattering signals and spectra. **a** The probe signal, with 30 scanning frequencies, covers CO_2_ and HDO absorption lines. **b** The reference signal without gas absorption. **c** The total optical depth spectra of CO_2_ and HDO at different ranges. **d** Lorentzian fitting of the range-resolved spectra; magenta dots are the measured ∆DOD values at 4 km with ∆*R* = 60 m

The raw backscattering signals of the probe light *N*_*i*_(*ν*_*i*_, *R*_*j*_) and the reference light *N*_0_(*ν*_0_, *R*_*j*_) are shown in Fig. 3a, b, respectively. The CO_2_ absorption feature is clear around the center frequency of *N*_*i*_(*ν*_*i*_, *R*_*j*_) at ~190.667 THz. From step 1 to step 3, the spectra of total optical depth DOD(*ν*_*i*_, *R*_*j*_) integrated over different ranges can be obtained, as shown in Fig. 3c. Then, in step 4, PDFS acquires the range-resolved spectra at different range cells. Fig. 3d illustrates one example of ΔDOD, at 4 km with a range resolution of 60 m. After step 5, the CO_2_ and HDO lines are separated by Lorentzian nonlinear fitting. Note that, the concentration of HDO varies with atmospheric temperature at different seasons, with a lower value in winter than that in summer^40^.

### Continuous observation

**Fig. 4:**
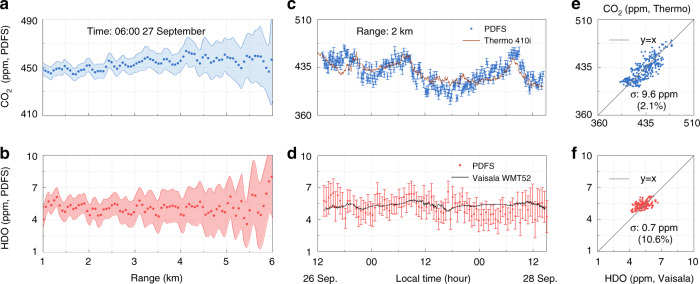
Comparisons and error analysis. **a** The range plot of CO_2_ concentrations at 6:00 on September 27, 2020, with a range resolution of 60 m. **b** The range plot of HDO concentrations. **c** The time plot of CO_2_ concentrations at 2 km, with a time resolution of 10 min. **d** The time plot of HDO concentrations, with a time resolution of 30 min. **e** Correlation between PDFS and Thermo Scientific 410i for CO_2_ measurements, with a standard deviation of ±2.1%. **f** Correlation of HDO measurements, with a standard deviation of ±10.6%. Shaded ranges and error bars indicate the 1*σ* standard deviation

Continuous field observation is carried out on the top of a building at the University of Science and Technology of China (USTC) (31.83°N, 117.25°E). To compare the results with in-situ sensors conveniently, the laser beam is emitted horizontally at a height of 50 m above ground level. In-situ CO_2_ analyzer (Thermo Scientific 410i) and humidity analyzer (Vaisala WMT52) are placed at the same height and 2 km away from the PDFS system in the laser path. The retrieved concentrations plotted versus range and time are shown in Fig. 4. The range resolution is 60 m, and the time resolutions for CO_2_ and HDO are 10 and 30 min, respectively. The measurement errors for both CO_2_ and HDO increase with the detecting range (Fig. 4a, b), due to SNR decaying along the range. And both concentrations of CO_2_ and HDO show good consistency with the results of the in-situ analyzers (Fig. 4c, d). The errors of HDO are larger than those of CO_2_, due to sparse frequency sampling and weak absorption of HDO over the optical frequency span. The averaged retrieval precisions at 2 km are ±5.4 and ±0.9 ppm, corresponding to ±1.2% and ±14.3% for the ambient concentration levels of CO_2_ and HDO, respectively. The comparisons between the in-situ analyzers and the PDFS measurements are plotted in Fig. 4e, f. The standard deviations of CO_2_ and HDO are ±9.6 and ±0.7 ppm, corresponding to ±2.1% and ±10.6%, respectively.

**Fig. 5:**
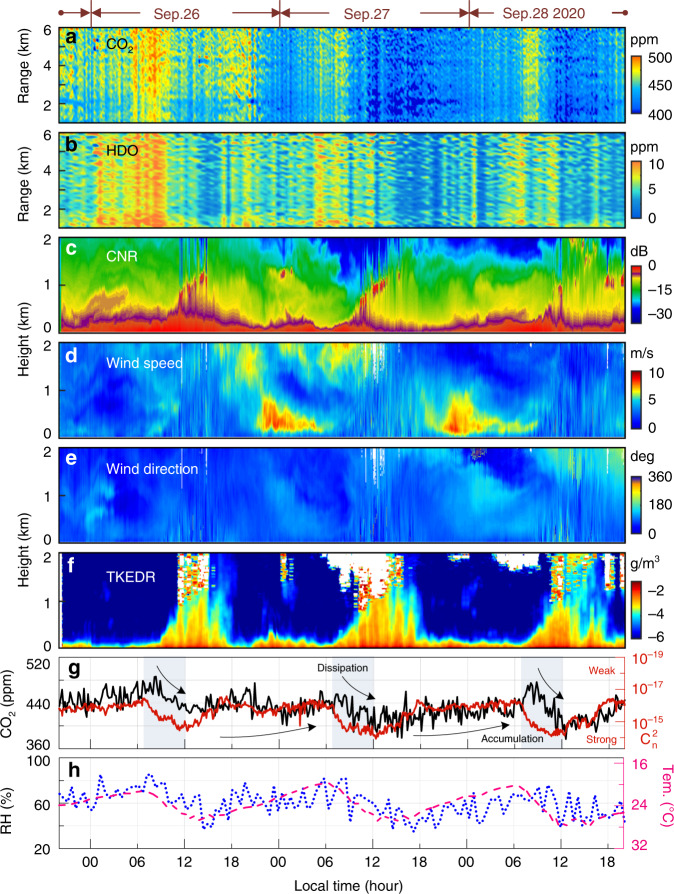
Results of continuous observation. Range-time plots of **a** CO_2_, **b** HDO. Height-time plots of **c** CNR, **d** Horizontal wind speed, **e** Horizontal wind direction, **f** Turbulent kinetic energy dissipation rate (TKEDR). **g** CO_2_ concentration and $C_{{n}}^2$. The black line represents the CO_2_ concentration at 2 km measured by PDFS. The red line is $C_{{n}}^2$ measured by a scintillometer, with the *y*-coordinate reversed. **h** Relative humidity (RH) and temperature (tem.) The magenta dotted line represents the RH at 2 km. The blue dashed line represents the temperature measured by Vaisala WMT52, with the *y*-coordinate reversed

Fig. 5a, b show the horizontal range-time distributions of the concentrations of CO_2_ and HDO measured by PDFS over 72 h. CO_2_ is unevenly distributed along with the range, affected mainly by the distribution of vegetation. Especially, in the ranges of 1.2–2.5 and 4.0–4.5 km, the CO_2_ concentration shows some lower marks during the daytime, which are caused by the photosynthesis of plants in the parks there. Beyond that, the CO_2_ and HDO concentrations show diurnal variation trends along the time and fluctuation along the range with fence-like patterns. Many factors may contribute to these phenomena, such as dissipation caused by turbulence, atmospheric transport, human activities, industrial production, etc. Thus, other instruments are employed to monitor the wind field and turbulence for further verifications.

A coherent Doppler wind lidar (CDWL) is used to monitor local meteorological conditions. The results are shown in Fig. 5c–f. The carrier-to-noise ratio^18^ (CNR, defined as the ratio of the mean radio-frequency signal power to the mean noise power) reflects the backscattering signal intensity from an aerosol. Fig. 5c shows that there are rarely external transmissions and no sudden fall of aerosol. In addition, Fig. 5d, e provides the horizontal wind speed and horizontal wind direction. It is noteworthy that the horizontal wind speed in the near ground region is <5 m/s, and the wind direction is mainly easterly. Considering that there is no industrial area in the east of the campus, the relatively stable wind field shows that CO_2_ and HDO are hardly affected by external transmission. The turbulent kinetic energy dissipation rate (TKEDR)^41^ in Fig. 5f shows that the local turbulence of the atmosphere is strong during the daytime. The diurnal variation of turbulence dominates the gas concentrations near the ground.

Fig. 5g shows the CO_2_ concentrations at 2 km measured via PDFS and the near-ground atmospheric refractive index structure constant $C_{{n}}^2$ measured by a large-aperture scintillometer (Kipp & Zonen LAS MKII). The scintillometer is placed on the top of a building with a height of 50 m, and its transmitter and receiver are located at a range of 1.1 and 2 km from the PDFS. For the convenience of readers, the *y*-coordinate of $C_{{n}}^2$ in Fig. 5g is reversed. During the continuous observation, the turbulence intensity gradually increases every morning from 8:00 to 12:00. Meanwhile, the CO_2_ concentration dissipates rapidly. Then, the turbulence intensity decreases in the afternoon and remains weak during the night, whereas the CO_2_ concentration accumulates gradually. On the one hand, the correlation between the turbulence intensity and the concentration of CO_2_ is shown clearly. The CO_2_ shows a diurnal variation along with the trend of $C_{{n}}^2$ throughout the whole observation time. On the other hand, there is a time delay between the trends of concentration and turbulence intensity, especially during the morning. Within the range of several kilometers, usually, the resolution of satellite payload equipment, the concentrations of CO_2_ and HDO change almost simultaneously. On the time scale, they are mainly affected by the atmospheric conditions of the boundary layer, especially the turbulence. Figure 5h shows the opposite trend in terms of the variation of relative humidity (RH) as measured by PDFS and the temperature measured by the in-situ sensor (Vaisala, WMT 52), where the *y*-coordinate of temperature is also reversed. The RH is retrieved from the measured HDO concentration using an empirical relative abundance between H_2_O and HDO. The natural abundance of H_2_O and HDO are 0.997317 and 0.000311, respectively^38^.

## Discussion

In conclusion, a PDFS method has been proposed and demonstrated for the remote sensing of multi-gas spectra at different locations over 6 km. To obtain precise spectra during scanning for open-path measurements, a stabilized probe laser frequency is provided by locking it to the optical comb. A reference laser is alternatively emitted with the probe laser using the time-division multiplexing technique, reducing the influences of aerosol variation, laser power fluctuation, detector instability, and telescope coupling efficiency change. Moreover, an SNSPD with a large active area, wideband response, and low dark noise is employed for long-range detection, making wideband PDFS possible with a low-power fiber laser. With further development, PDFS can be updated to measure the distributed spectra over C and L bands, so that abundant gases, such as CO, CO_2_, H_2_O, HDO, NH_3_, and C_2_H_2_ can be analyzed within a single system (Table S1). Future applications of PDFS include long-range warnings of flammable, explosive and toxic substances, and monitoring of industrial pollution. Furthermore, substance evolution and chemical reactions in the atmosphere can be further investigated.

## Materials and methods

### The principle of comb-referenced frequency locking

**Fig. 6:**
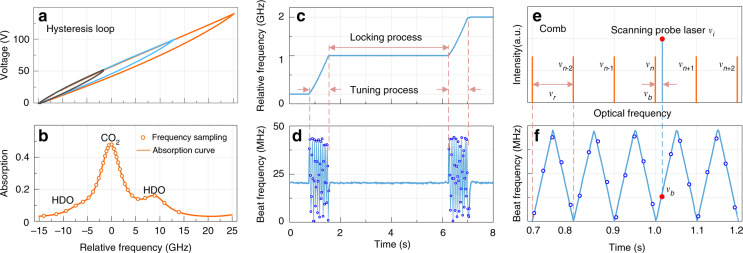
Principle of comb-referenced frequency-locking technique. **a** The hysteresis loops show the mapping between frequency and voltage of the probe laser. The lines in different colors show the loops for controlling voltage in different ranges. **b** The absorption functions of CO_2_ and HDO from HITRAN. The hollow circles represent the frequency steps used to sample the whole line shape. **c** The relative optical frequency variation versus time. **d** The beat frequency variation versus time. The locking process is shown in the flat lines, and the tuning process is shown in the triangle-wave-like track. **e** The probe laser *ν*_*i*_ beats with the optical comb *ν*_n_, mapping optical the frequency into radio frequency signals. **f** Detailed description of the tuning process. As the probe laser beats with the nearest frequency comb teeth one by one, the beat frequency *ν*_b_ varies according to a triangle-wave-like track

Figure 6 shows the principle of comb-referenced frequency scanning and locking technique^40^. The probe ECDL is tuned by controlling its piezo-electric transducer (PZT). The hysteresis loops of voltage versus relative optical frequency are shown in Fig. 6a. Different colors of loops represent different dynamic ranges of the PZT. In the scanning range of 190.652–190.682 THz, one CO_2_ absorption line and two weak HDO lines are found in a ground-level atmosphere condition according to HITRAN 2016 (ref. ^38^). As shown in Fig. 6b, the frequency in the *x*-coordinate is relative to the center frequency 190.667 THz of the CO_2_ absorption feature. The 30 orange hollow circles represent the non-uniform frequency steps, which are used to sample the mixture spectrum. Denser frequency steps are used in the CO_2_ line for better precision in the measurement of CO_2_, making HDO an additional result. It is worth noting that the measurement precision of HDO can be improved by changing the scanning numbers and intervals (different frequency sampling modes are shown in Fig. S4).

The frequency of the probe laser (*ν*_*i*_) is locked to the comb through a feedback locking loop^27,28^. As shown in Fig. 1, an optical frequency comb with a repetition rate *ν*_r_ = 100 MHz and carrier-envelope offset frequency *ν*_ceo_ = 20 MHz are referenced to a rubidium clock. A tiny portion (~1%) of the probe laser and the comb are coupled into a balanced detector. The beat frequency between the probe laser and the nearest frequency comb tooth is selected by using a low-pass filter (LPF) with a bandwidth of 48 MHz, which is slightly lower than half of the repetition rate of the comb (100 MHz). Then the filtered beat frequency is read via a frequency counter (Keysight 53230A) and recorded on the computer. In the locking process (Fig. 6c, d), the beat frequency (*ν*_b_) is compared with a reference frequency of 20 MHz, then the difference frequency signal is fed back to adjust the voltage of the PZT in the probe laser. Finally, the probe frequency is locked to the comb tooth with an offset frequency of 20 MHz. Note that the comb is a stabilized frequency ruler (*ν*_ceo_ reaches frequency stability of 1.2 × 10^−9^ in an integration time of 1 s), the fluctuation of the beat frequency is dominated by the uncertainty of the probe frequency. The standard deviation of the locked probe frequency is 0.5 MHz in an integration time of 120 s. During the tuning process, the relative frequency of the probe laser ramps with time (Fig. 6c). As shown in Fig. 6e, f, the probe laser beats with the nearest frequency comb teeth one by one in the frequency domain, generating a triangle-wave-like track for the beat frequency. The peak beat frequency of the triangle-wave-like track is restrained in *ν*_r_/2 using the LPF. While the probe laser scans from one comb tooth to another adjacent tooth, the beat frequency goes through one period. Thus, the absolute frequency interval between the frequency steps can be calculated from the track of the recorded beat frequency. The minimum frequency interval is limited by the repetition frequency of the comb.

### Triple-peak Lorentzian nonlinear fitting

Since the experiment is carried out near the ground, we can assume that the Doppler line shift is negligible^36,42^. Therefore, a Lorentzian line shape can be used to fit the obtained spectrum. The Lorentzian model can be expressed as

5 $$\varphi \left( \nu \right) = \frac{{2A}}{\pi }\frac{{\omega _{{{\mathrm{L}}}}}}{{\omega _{{{\mathrm{L}}}}^2 + 4\left( {\nu - \nu _{{{\mathrm{c}}}}} \right)^2}}$$

where *A* = *Hω*_L_*π*/2 is the area of Lorentzian line shape, *H* represents the height of the line center, *ν*_c_ is the center frequency of probe laser, and *ω*_L_ is the FWHM, which is determined by^36^

6 $$\omega _{{{\mathrm{L}}}} = 2\gamma _0\left( {\frac{P}{{P_0}}} \right)\left( {\frac{{T_0}}{T}} \right)^{n_{\mathrm {t}}}$$

here *P* and *T* are the ambient pressure and temperature, *γ*_0_ is the pressure broadening coefficient at *P*_0_ = 1 atm and *T*_0_ = 273 K, and *n*_t_ is the temperature exponent. The mixture line shape of CO_2_ and HDO is the superposition of three Lorentzian models, the independent line shape of each molecule can be obtained by performing a triple-peak Lorentzian fitting. Meanwhile, the fitted Lorentzian area of the range-resolved differential absorption depth can be expressed as

7 $$A = \mathop {\int}\limits_{{\mathrm {line}}} {{\Delta}{\mathrm {DOD}}\left( \nu \right)} {{{\mathrm{d}}}}\nu = S\left( T \right)N_{{{\mathrm{d}}}}L$$

where *L* is the length of the range cell, *N*_d_ is number density, and *S*(*T*) is the spectral line intensity which can be written as^36^

8 $$S\left( T \right) = S_0\left( {\frac{{T_0}}{T}} \right)^{3/2}\left\{ {\frac{{1 - \exp \left[ {hc\nu _{{{\mathrm{c}}}}/k_{{{\mathrm{B}}}}T} \right]}}{{1 - \exp \left[ {hc\nu _{{{\mathrm{c}}}}/k_{{{\mathrm{B}}}}T_0} \right]}}} \right\} \times \exp \left[ {\frac{{E^{\prime\prime}hc}}{k}\left( {\frac{1}{{T_0}} - \frac{1}{T}} \right)} \right]$$

here *S*_0_ is the line strength at *P*_0_ = 1 atm and *T*_0_ = 273 K, *h* is the Planck constant, *c* is the speed of light, *k*_B_ is the Boltzmann constant, *E*″ is the lower-state energy. By combining Eqs. (5)–(8), the concentration (*χ*) of the molecule under investigation is retrieved as

9 $$\chi = \frac{A}{{S\left( T \right)Ln_{{{\mathrm{L}}}}}}\frac{{P_0}}{P}\frac{T}{{T_0}} \times 10^6$$

where *n*_L_ = *P*_0_/(*k*_B_·*T*_0_) = 2.688256 × 10^25^ molecule/m^−3^.

## Supplementary information

Supplementary information
