# Supplementary material for: Photon-counting distributed free-space spectroscopy
Source: Light Sci Appl. 2021 Oct 12;10:212. doi: 10.1038/s41377-021-00650-2 (PMC8511071; doi:10.1038/s41377-021-00650-2)
Supplement: Supplementary file 1 — Supplementary information [file 41377_2021_650_MOESM1_ESM.doc]

**Supplementary Information for**

**Photon-counting Distributed Free-space Spectroscopy**

Saifen Yu^1,2★^, Zhen Zhang^1,2★^, Haiyun Xia^1,2,3🖂^, Xiankang Dou^1,3^, Tengfei Wu^4^, Yihua Hu^5^, Manyi Li^1^, Mingjia Shangguan^6^, Tianwen Wei^1^, Lijie Zhao^1^, Lu Wang^1^, Pu Jiang^1^, Chengjun Zhang^7^, Lixing You^7^, Leigang Tao^3^, Jiawei Qiu^2^

^1^ School of Earth and Space Science, University of Science and Technology of China, Hefei 230026, China

^2^ School of Atmospheric Physics, Nanjing University of Information Science &Technology, Nanjing 210044, China

^3^ Hefei National Laboratory for Physical Sciences at the Microscale, Heifei 230026, China

^4^ Changcheng Institute of Metrology & Measurement, Aviation Industry Corporation of China, Beijing 100095, China

^5^ State Key Laboratory of Pulsed Power Laser Technology, National University of Defense Technology, Hefei 230037, China

^6^ State Key Laboratory of Marine Environmental Science, College of Ocean and Earth Sciences, Xiamen University, Xiamen, 361102, China

^7^ Shanghai Institute of Microsystem and Information Technology, Chinese Academy of Sciences, Shanghai 200050, China

^★^These authors contributed equally: Saifen Yu, Zhen Zhang

^🖂^Correspondence: *Haiyun Xia (*[*hsia@ustc.edu.cn*](mailto:hsia@ustc.edu.cn)*)*

1 Absorption line selection

The selection of appropriate absorption lines is ruled by a series of constraints^1^: (1) The selected absorption spectrum should avoid interference from other atmospheric molecules. (2) The line should be characterized by a suitable optical depth. A weak optical depth results in low absorption. On the contrary, a strong absorption deteriorates the transmission, making the received lidar signal weak. (3) The line should be temperature insensitive, minimizing the effect of temperature fluctuation along the optical path.

1.1 Absorption line interference

One can select the absorption line with the lowest interference according to the HITRAN 2016 database^2^. When measuring the spectrum of CO_2_, the main interference gas is H_2_O (ref. 1). Figure S1 shows the absorption cross sections of CO_2_, H_2_O and HDO in the range of 185.185–196.078 THz, which are corrected by natural abundance. The R16 line of CO_2_ is preferred where the absorption of H_2_O is very low. Note that, the R16 line overlaps the absorption of HDO, which is usually neglected in other researches. Here, both spectra of CO_2_ and HDO are recorded simultaneously. The choice is conductive to demonstrate the capability of PDFS for multi-gas detection.


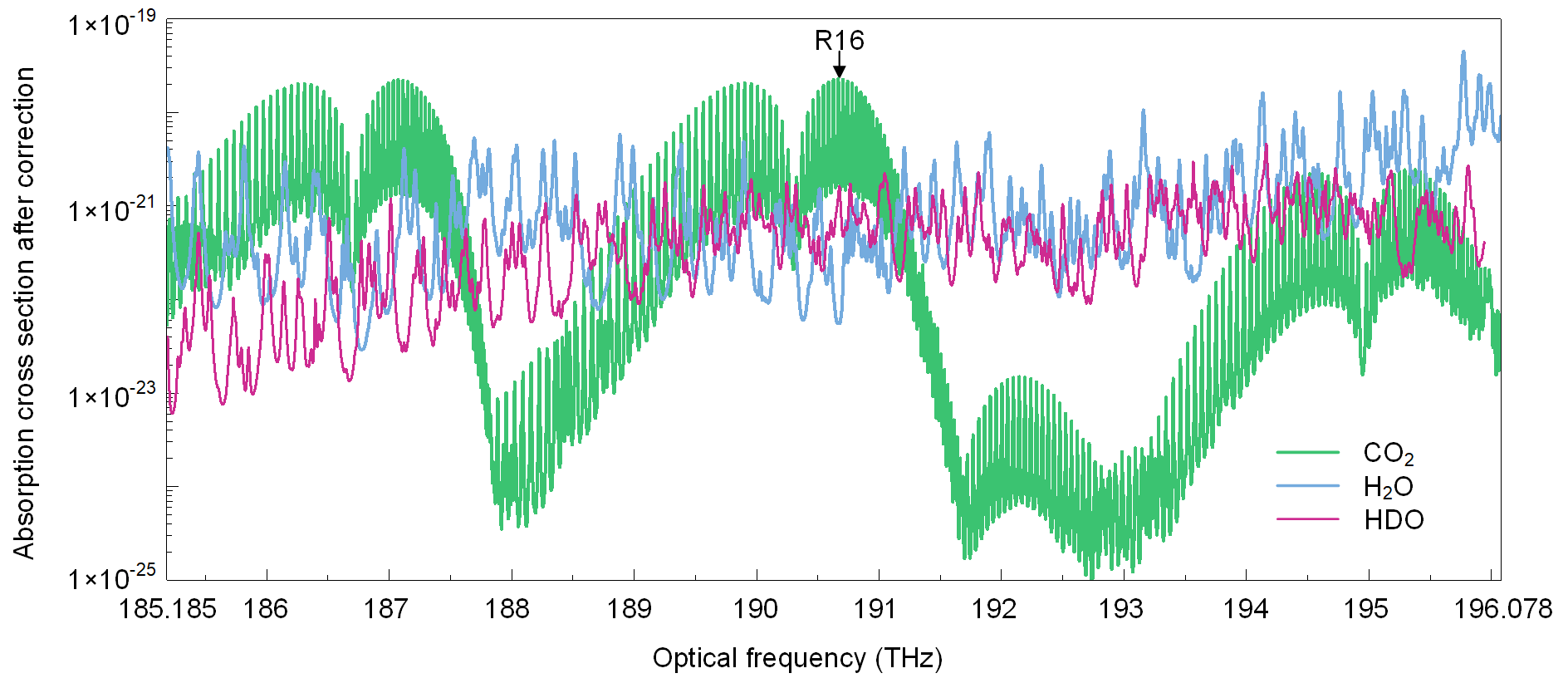


Figure S1| The absorption cross section of CO_2_, H_2_O, and HDO from HITRAN. The cross section is corrected by natural abundance.

1.2 Optical depth optimization

A priori determination of the optical depth is evaluated by characterizing the number density error within a range bin. For a system where the main source of random noise is shot noise, the error can be estimated as^1,3^

$\frac{\delta N_{\text{d}}}{N_{\text{d}}}=\frac{K_{OD}}{OD\sqrt{m_{0}}\left(SNR\right)}\times\left\{\frac{\exp\left(2OD\right)\left[1+\exp\left(2OD/K_{OD}\right)\right]}{2}+1\right\}^{1/2}$ (S1)

where *N*_d_ is the number density, *K_OD_* is the ratio between the optical depth (*OD*) and the optical depth in a range bin (Δ*OD*), *m*_0_ is the total number of laser shots. Fig. S2 shows the function of the error and the optical depth under different SNR.


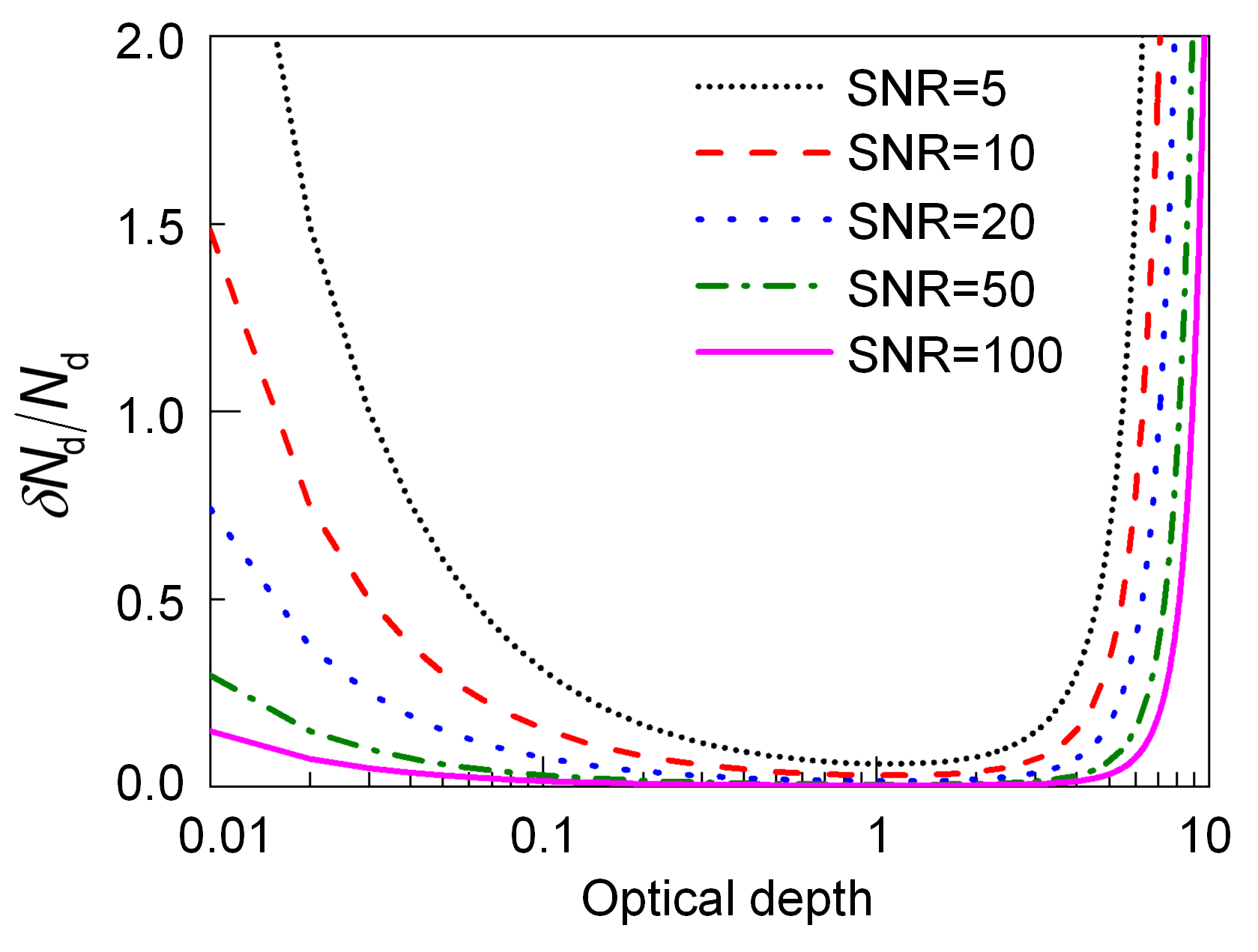


Figure S2| The function of the number density error and the optical depth. Here, *m*_0_ is assumed as 4×10^5^, and the optical depth of 2 km and the range bin of 60 m correspond to *K_OD_*≈33.

On the one hand, the error of number density increases as the SNR decreases. On the other hand, the error becomes larger when optical depth *OD*>4 or *OD*<0.02. The minimum error occurs at an optical depth *OD*=1.1.

The optical depth is not only related to the line strength, but also related to the concentration of the molecule under investigation. The optical depth at the peak of the absorption line can be determined by Eq. (5)–(9) as

$OD=2\chi L\frac{P}{P_{0}}\frac{T_{0}}{T}\frac{S\left(T\right)\cdot n_{\text{L}}}{\pi\omega_{\text{L}}}\times10^{-6}$ (S2)

where *L* is length of the optical path, *P* and *T* are the ambient pressure and temperature, *P*_0_=1 atm and *T*_0_=273 K, *n*_L_=*P*_0_/(*k_B_*·*T*_0_)=2.688256×10^25^ molecule/m^-3^. *ω*_L_ is the FWHM of the line shape described in Eq. (6). *S*(*T*) is the line strength described in Eq. (8).

Therefore, the optimum line strength of the selected line can be calculated with an expected concentration and the optimum optical depth. And the error for various molecules at different line strengths can be analyzed from Eq. (S2).

For example, assume that the ambient concentration of CO_2_ is 450 ppm, the optimum line strength should in the range of 2.15×10^-24^–4.25×10^-22^ cm^-1^/(molecule·cm^-2^). Here, the selected CO_2_ R16 line at 190.667 THz shows a relatively higher line strength of 1.779×10^-23^ cm^-1^/(molecule·cm^-2^) than other CO_2_ lines in the C and L bands^2^. According to the Equation above, the expected error for CO_2_ and HDO concentration measurement at 2 km with a 60 m spatial resolution is 0.9% and 8.4% for the ambient levels.

1.3 Temperature dependence

The temperature dependence of the absorption line can be determined from the derivative of absorption coefficient *α*_g_ with respect to temperature^4^

$\frac{d\alpha_{\text{g}}}{\alpha_{\text{g}}}=\frac{\text{d}T}{T}\left[\begin{matrix} \frac{\varepsilon hc}{k_{\text{B}}T} \end{matrix}-\frac{5}{2}+\Xi\right]$ (S3)

where *ε* is the ground state energy of the transition available from the database, and Ξ is a value dependent on the line shape, which is zero for pure Doppler broadening and one for pure Lorentzian broadening. Since the experiment is carried out near the ground, where the lines of molecular bands are usually almost pure Lorentzian^1^, Ξ=1 is used for the calculations. So the absorption line becomes sensitive to temperature as the ground state energy increases.


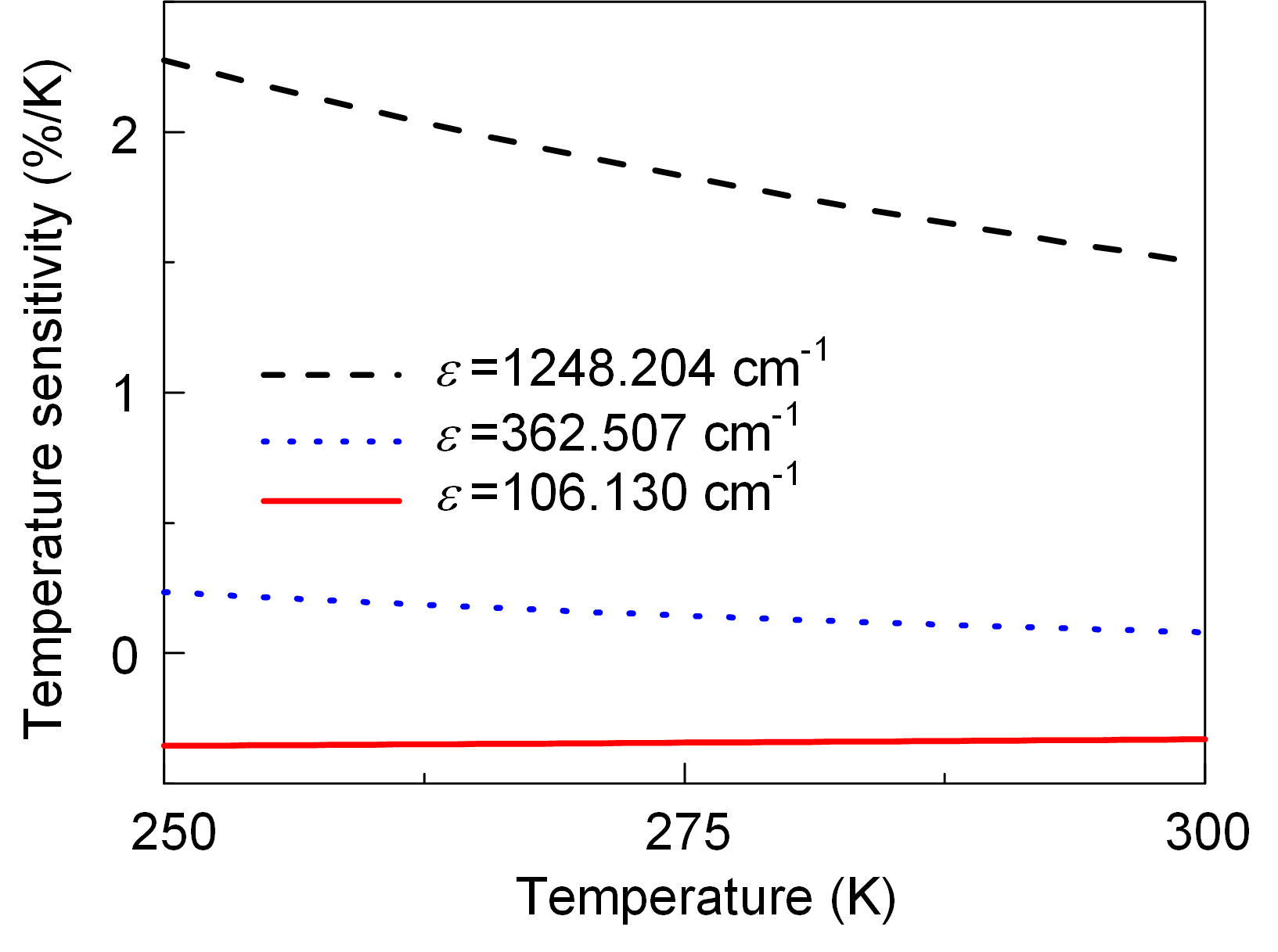


**Figure S3| The temperature sensitivity as a function of temperature.** Lower ground state energy corresponds to lower temperature sensitivity.

Here, the R16 line of CO_2_ is temperature insensitive with a ground state energy of 106.130 cm^-1^. The ground state energies of two HDO line are 100.391 cm^-1^ and 362.507 cm^-1^, respectively. For temperature measurement, a temperature sensitive line such as O_2_ absorption line at 389.729 THz with a ground state energy of 1248.204 cm^-1^ is usually used^4^. The comparison of the temperature sensitivity of these lines is shown in Fig. S3.

1.4 Outlook for measuring other gases

The tunable spectrum range of the probe laser is 185.185–196.078 THz. The parameters of several molecule absorption lines^1^ locating in this range are summarized in Table S1, where the abundance in atmosphere is considered in the line strength. Optimum concentration limit means the minimum concentration required when the optical depth *OD*>0.02 using the selected line strength. The concentrations of carbon dioxide (CO_2_) and vapour (H_2_O) in ambient of the atmosphere can be measured with low error, due to their ambient concentrations are greater than the lower limit of optimum concentration. The ambient concentration of carbon monoxide (CO) is 1.5 ppm while the value increases to 70 ppm (ref. 1) in the urban polluted air, which is close to the optimum limit. So that CO is also expected to be measured with a low error. For other gases such as ethyne (C_2_H_2_) and ammonia (NH_3_), whose ambient concentration is particularly low, the measurement can be performed in leaking case only.

**Table S1: The parameters of several molecule absorption lines**

| **Molecules** | **Wavenumber**  cm^-1^ | **Frequency**  THz | **Line strength**  cm^-1^/(molec·cm^-2^) | **Ground state energy**  cm^-1^ | **Ambient conc.**  ppm | **Optimum conc. limit**  ppm |
| --- | --- | --- | --- | --- | --- | --- |
| CO_2_ | 6359.967 | 190.667 | 1.808×10^-23^ | 106.130 | 420 | 55 |
| H_2_O | 6443.085 | 193.159 | 8.191×10^-25^ | 1006.116 | 18600 | 1170 |
| HDO | 6360.278 | 190.681 | 2.774×10^-22^ | 100.391 | 6 | 4.5 |
| CO | 6377.407 | 191.190 | 2.251×10^-23^ | 107.642 | 1.5 | 70 |
| C_2_H_2_ | 6574.361 | 197.094 | 1.333×10^-20^ | 65.887 | 1×10^-4^ | 0.9 |
| NH_3_ | 6568.307 | 196.913 | 2.571×10^-21^ | 85.068 | 3.6×10^-3^ | 0.45 |

2 Instrument description

**Table S2: The system parameters of the instruments**

|  | Parameter | Specification | Manufacturer and model |
| --- | --- | --- | --- |
| ECDL | Spectrum range | 1530.000–1620.000 nm  (185.185–196.078 THz) | Toptica, CTL1550 |
| Reference laser | Frequency | 190.652 THz | Rayshining, PEFL |
| Amplifier | Spectrum range | 1572.212–1572.459 nm  (190.652–190.682 THz) | Rayshining, EYDFA |
|  | ASE suppression ratio | 45 dB |  |
|  | Pulse repetition rate | 20 kHz |  |
|  | Pulse energy | 40 μJ |  |
|  | Pulse width | 400 ns |  |
|  | Power Stability | ≤5% rms |  |
|  | Linewidth | <2 MHz |  |
| Telescope | Diameter | 256 mm | Rayshining |
| Optical filter | Bandpass width | 0.3 nm flattop, peak transmittance>85% | Optizone, MMF1572 |
| Detector | Type | SNSPD | SIMIT |
|  | Active-area diameter | 50 μm |  |
|  | Quantum efficiency | 31.5% |  |
|  | Dark count (per second) | 100 |  |
|  | Noise Equivalent Power | 5.7×10^-18^ W/Hz^0.5^ |  |
| Comb | Pulse repetition rate and Allan deviation in 1s | 100 MHz | AVIC |
|  |  | 5×10^−12^ |  |
|  | Carrier-envelope offset and Allan deviation in 1s | 20 MHz |  |
|  |  | 1.2×10^−9^ |  |
| PDFS performance | Distance | 6 km | USTC |
|  | CO_2_ precision | 1.2% |  |
|  | HDO precision | 14.3% |  |
|  | Time resolution | 10 min |  |
|  | Range resolution | 60 m |  |

3 Frequency sampling mode

The sampling number is set as 30, which is sufficient to cover the entire spectrum. In this experiment, in order to analyze CO_2_ with a higher precision, non-uniform sampling interval is used, as shown in Fig. S4. The slopes on the leading and trailing edges of an absorption line are high, while the slopes in center and wings are low. Thus, small frequency sampling interval is used to record the edges of the CO_2_ line (Fig. S4a), leading to a lower fitting standard deviation of CO_2_ than that of uniform sampling results (Fig. S4b). Note that, the uniform mode in Fig. S4b shows small frequency sampling intervals at the wings of the line, which is beneficial to HDO measurement compared to Fig. S4a.

**
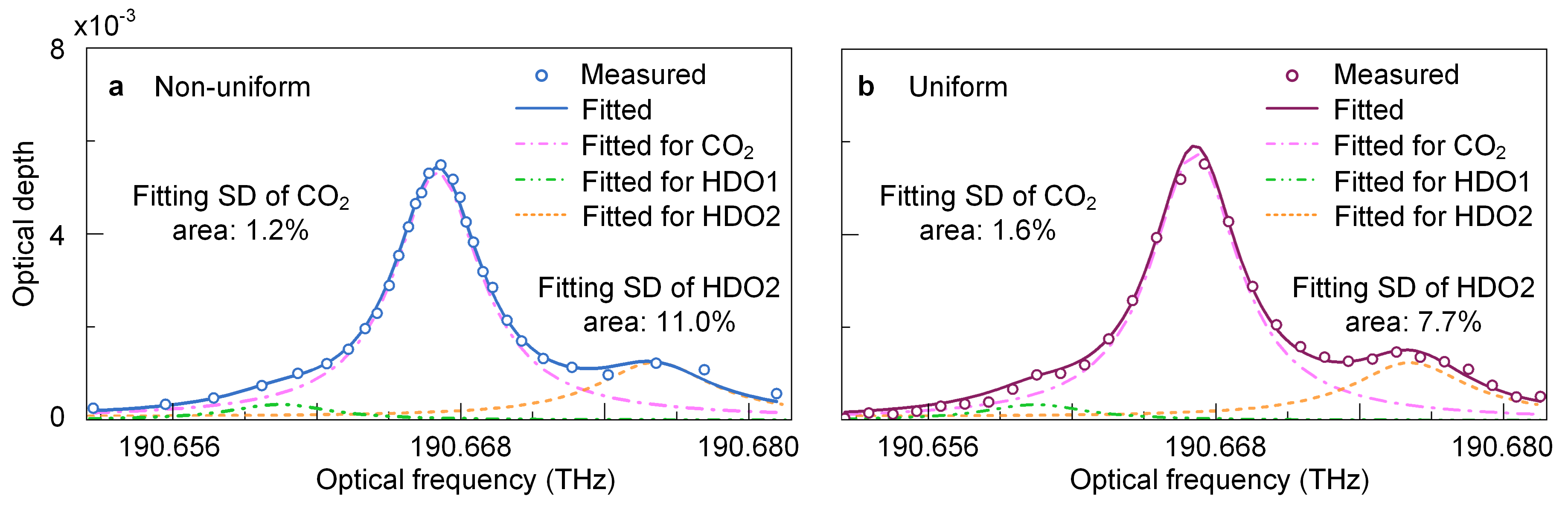
**

Figure S4| Comparison between non-uniform and uniform sampling mode.

**References**

1. Ambrico, P. F. *et al*. Sensitivity analysis of differential absorption lidar measurements in the mid-infrared region. *Applied Optics* **39**, 6847-6865 (2000).
2. Gordon, I. E. *et al*. The HITRAN2016 molecular spectroscopic database. *Journal of Quantitative Spectroscopy and Radiative Transfer* **203**, 3-69 (2017).
3. Megie, G. & Menzies, R. T. Complementarity of UV and IR differential absorption lidar for global measurements of atmospheric species. *Applied Optics* **19**, 1173-1183 (1980).
4. Theopold, F. A. & Bösenberg, J. Differential absorption lidar measurements of atmospheric temperature profiles: theory and experiment. *Journal of Atmospheric and Oceanic Technology* **10**, 165-179 (1993).
